# Early finding of chest wall metastasis of hepatocellular carcinoma in a woman by fluorodeoxyglucose-positron emission tomography scan: a case report

**DOI:** 10.1186/1752-1947-5-147

**Published:** 2011-04-13

**Authors:** Lixin Yang, Howard Marx, Yun Yen

**Affiliations:** 1 Department of Medical Oncology, City of Hope National Medical Center, 1500 East Duarte Road, Duarte, CA 91010, USA; 2 Department of Radiology, City of Hope National Medical Center, 1500 East Duarte Road, Duarte, CA 91010, USA

## Abstract

**Introduction:**

The use of fluorodeoxyglucose-positron emission tomography to evaluate well-differentiated hepatocellular carcinomas is facing critical problems. It is reported that the activity of fluorodeoxyglucose-6-phosphatase, which converts fluorodeoxyglucose-6-phosphatase to fluorodeoxyglucose, is high in normal liver cells. However, the enzyme-converting activity of glucose-6-phosphatase of well-differentiated hepatocellular carcinomas is similar to normal liver tissue. Thus, using fluorodeoxyglucose in diagnosing primary hepatocellular carcinomas is difficult. However, using fluorodeoxyglucose to detect extrahepatic metastasis of hepatocellular carcinomas is still possible.

**Case presentation:**

We describe the case of a 45-year-old Chinese woman who developed a recurrent lesion in the chest wall from a previous surgically resected hepatocellular carcinoma. This recurrent lesion was detected first on the basis of a positron emission tomography scan, then on the basis of a computed tomography scan and other clinical tests.

**Conclusion:**

This finding indicates that the positron emission tomography scan is a potentially reliable tool to screen for systemic metastatic disease in patients with hepatocellular carcinomas when other cross-sectional imaging tests such as computed tomography or magnetic resonance imaging are negative.

## Introduction

Hepatocellular carcinoma (HCC) is the fifth most common cancer worldwide [1]. It has a high mortality rate, with most patients succumbing to the disease within one year [1]. The presence of metastatic disease in these patients markedly reduces survival [2]. Recently, the occurrence of liver cancer in the United States of America has been increasing because of the increased frequency of hepatitis C [3]. Liver cancer tends to spread to nearby lymph nodes, bones and lungs. In rare cases, it spreads to the chest wall with or without identified primary liver cancer. It is essential to diagnose HCC in its early stages to ensure patient survival [2,4]. Ultrasound, computed tomography (CT), magnetic resonance imaging (MRI) and positron emission tomography (PET) are the primary imaging methods for the diagnosis of liver cancer [5]. The PET scan is an emerging imaging modality that measures and distinguishes the increased rates of glucose metabolism and oxygen consumption in tumor tissue from normal surrounding tissue. The advantage of PET scans is that they measure metabolic changes at the cellular level [6]. On the other hand, cross-sectional imaging modalities such as CT, ultrasound, and MRI detect anatomical changes. Thus, PET scans allow complementary anatomical detection and measurement of cellular activity in tumors. However, the average false-negative rate of PET scans is 40% to 50% for the detection of HCC because of active glucose metabolism in normal hepatic parenchyma. It has been demonstrated that glycolysis metabolism within HCC is equivalent to or less than that of normal liver tissue. Therefore, most primary HCCs are underdetected by PET scans [7,8]. However, PET scans have shown benefit in the detection of early metastasis in extrahepatic sites, especially in the frequent metastatic organs, such as the colon, lung, brain and lymph nodes [9,10]. In this case report, we describe a chest wall metastasis of HCC that was uniquely detected by PET scan but not by CT scan. This report indicates that whole-body PET is a potentially valuable method to screen for the early presentation of extrahepatic metastatic disease.

## Case presentation

A 45-year-old Asian woman with a known history of hepatitis C for 20 years had received no interferon treatment for her hepatitis C. The patient developed HCC and underwent successful surgical resection with a negative surgical margin. Her pathological specimen allowed the diagnosis of well-differentiated HCC in postsurgical follow-up, and the patient underwent CT scans every three months, alternating with ultrasound. All results of these scans were reported to be unremarkable except for postsurgical changes. A blood test was also done, including measurement of α-fetoproteins (AFPs). One year later the patient's AFP was slightly elevated at 25 mg/dL (normal range, <10 mg/dL). Repeated CT scans and ultrasounds of the patient's abdomen revealed no changes from previous examinations. Upon physical examination, the patient was noted to have small induration areas over the upper-left chest wall and under the left clavicle bone with a size of approximately 1 × 1 cm^2^. The patient denied any trauma or pain related to the area. Because AFP levels continued to increase to 40 mg/dL, a PET scan was ordered. For PET imaging, the patient was injected with 10.9 mCi of ^18^F-fluorodexyglucose (FDG). One hour later the patient voided and underwent PET with or without attenuation correction. Imaging was performed in multiple acquisitions to include the face, neck, chest, abdomen, pelvis and proximal thighs. Images were acquired using 2-D imaging and reconstructed in three planes. 3-D tomographic reconstruction and analysis were carried out.

**Figure 1:**
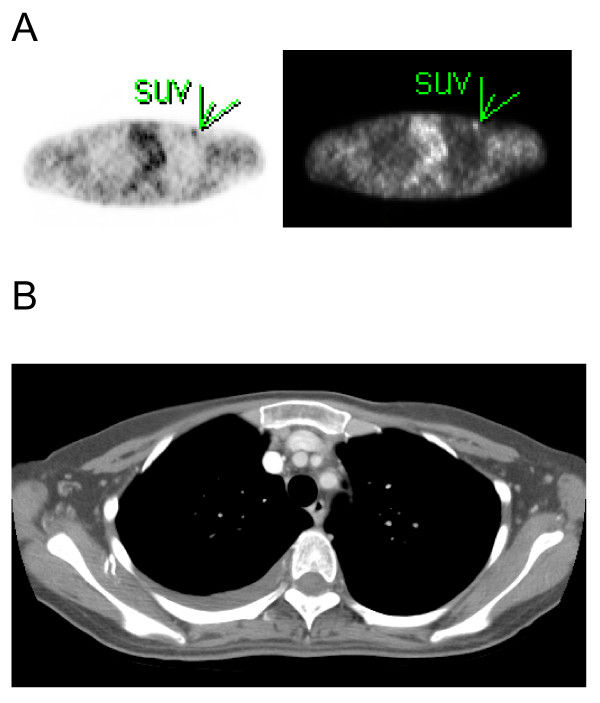
**(a) Metastatic lesion in chest wall was markedly positive for uptake with tracer (arrow)**. (b) The corresponding computed tomographic image reveals that no lesion can be identified.

The PET scan showed a focus of slightly elevated FDG uptake in the left infraclavicular region with a standardized uptake value of 2.0 (Figures 1a and 1b). It was interpreted as showing possible metastatic disease. However, the CT scan was interpreted as showing no residual tumor or metastatic disease. Subsequently, the patient underwent resection of a palpable subcutaneous nodule not visualized on the CT scan, but correlated with the above-described PET scan abnormality in the left infraclavicular region. The patient's pathological examination confirmed metastatic HCC of the chest wall.

## Discussion

HCC is the third highest cause of cancer mortality worldwide, and the incidence of HCC in the United States is increasing [4]. The etiology of HCC is believed to be from hepatitis B and C, but its increased incidence is due to metabolic disorders such as diabetes. The diagnosis of HCC is dependent upon AFP, tissue biopsy and imaging studies. Although ultrasound, CT and MRI have been widely used to detect HCC, PET has remained out of favor for the diagnosis of HCC because of the lack of differentiation between HCC glucose metabolism and normal liver tissue. However, on the basis of a literature search, the PET scan has shown promise in distinguishing extrahepatic metastatic tumors from normal surrounding tissue. The reports show that the sensitivity of PET is about 80%, its specificity is 75% to 90% and its accuracy is around 80% [9,10]. Thus, PET may detect early extrahepatic HCC lesions in clinical follow-up. In the reported studies, the PET scan has been successfully used in detecting extrahepatic HCC lesions such as those of the lymph node, lung, bone, vasculature and adrenal gland. However, some uncommon metastatic sites of HCC, such as skin or soft tissues, have not been detected by PET or have not been reported yet. On the other hand, lesions in these tissues can be missed by using CT or MRI technologies. The PET scan, by measuring elevated glucose metabolism in tumors, has shown promise in distinguishing extrahepatic metastatic tumors from normal surrounding tissue [9,10]. Thus, the use of PET in clinical follow-up may be beneficial in detecting early metastatic HCC lesions which are not defined in the liver.

In the patient described in the present report, the chest wall tumor that developed from HCC was detected by obtaining a PET scan as the earliest step, in comparison to a CT scan. This result provided very valuable and unique information that affected the patient's treatment plan. The patient underwent immediate local resection of the metastatic lesion in accordance with the standard treatment guideline [11]. Furthermore, the patient was started on sorafenib treatment as a first-line targeted therapy immediately after the PET scan identified the metastatic lesion over the chest wall. The response to sorafenib treatment provides further progression-free survival. Therefore, the early diagnosis of a metastatic lesion on the basis of a PET scan ensures a better prognosis for the patient, initiation of the most accurate therapy and increased patient survival. Although most HCC metastases occur in the gastrointestinal tract, lung and bone, HCC does metastasize to many other organs, including the chest wall [12]. Therefore, it is important to be able to identify these lesions in an imaging study such as a PET scan, as in our present case, where PET was the only tool that successfully detected a pathologically proven chest wall metastasis. Although further investigation is needed with larger groups of patients to validate the superiority of PET to detect extrahepatic metastatic disease in patients with HCC, we believe it is a promising method, in combination with other studies, to detect rare sites for metastatic disease. Even though the PET scan is regarded as an insensitive method for the detection of primary HCC, one clinical study did demonstrate that ^18^F-FDG PET/CT had a relatively high sensitivity in detecting extrahepatic HCC lesions [13].

## Conclusion

It has been reported that the PET scan provides additional information for the diagnosis of extrahepatic HCC [9,14] and is an important tool for surveillance of distant metastasis in patients with unexplained AFP elevation after HCC treatment [15]. Therefore, we suggest that the use of whole-body ^18^F-FDG PET/CT be considered in the follow-up of patients with HCC for potential early detection of systemic metastatic extrahepatic disease.

## Abbreviations

AFP: α-fetoprotein; CT: computed tomography; ^18^F-FDG: ^18^F-fluorodeoxyglucose; GI tract: gastrointestinal tract; HCC: hepatocellular carcinoma; MRI: magnetic resonance imaging; PET: positron emission tomography; SUV: standardized uptake value.

## Competing interests

The authors declare that they have no competing interests.

## Consent

Written informed consent was obtained from the patient for the publication of this case report. A copy of the written consent is available for the review by the Editor-Chief of this journal.

## Authors' contributions

All authors contributed to the writing and editing the case report. LY carried out the literature search. YY collected the medical history. HM obtained the images. All the authors read and approved the final manuscript.
